# An Investigation into The Role of Lifestyle Factors in SuperAgers

**DOI:** 10.1002/alz.088812

**Published:** 2025-01-09

**Authors:** Philippa Watson, Ivan Koychev, John Gallacher, Sarah Bauermeister

**Affiliations:** ^1^ University of Oxford, Oxford, Oxon United Kingdom

## Abstract

**Background:**

It is widely acknowledged that cognitive abilities tend to decline with age; however, there exists considerable intra‐individual variability in the rate of decline. While many individuals report varying degrees of cognitive decline, a small subset known as “SuperAgers” exhibits cognitive abilities comparable to those several decades younger. Our understanding of which factors enable SuperAgers to maintain their superior cognitive abilities is inconclusive. It is hypothesized, however, that the factors safeguarding against cognitive decline may also play a role in maintaining exceptional cognitive abilities.

**Method:**

This study leverages data collected during Phase 5 (1997‐1999) and Phase 12 (2015‐2016) of the Whitehall II cohort, a longitudinal study focusing on British civil servants. SuperAgers were defined as those over 65 years old at Phase 12 who scored in the top tercile for verbal memory performance in Phase 12 and who performed above the mean for verbal memory at Phase 5. Structural equation modelling will be employed to conduct a multivariate statistical analysis investigating the association between engagement in various lifestyle factors (e.g., mental health, physical activity, social engagement, and biomedical factors) in SuperAgers and cognitively normal agers (CN’s).

**Result:**

The study comprised 1,963 participants (511 females), with 812 meeting the criteria for SuperAgers and 1,156 for CN’s. SuperAgers exhibited lower levels of hypertension (*t* = 5.13, *p* < .001), medications (*t* = 2.86, *p* = .002), BMI (*t* = 1.79, *p* = .04), and higher engagement in high social activities (*t* = 3.44, *p* < .001), low social activities (*t* = 2.90, *p* = .002), and vigorous physical activities (*t* = ‐2.00, *p* = .02). No significant differences were observed between SuperAgers and CN’s regarding smoker status, alcohol consumption, sleep duration, mental health, and cardiovascular diseases (*p* > .05). Additional analyses are ongoing and will be completed by the conference date.

**Conclusion:**

The findings from this study will provide valuable insights into the contribution and significance of various modifiable lifestyle factors in preserving cognitive function during older age.

